# 3D registration-guided deformable residual inpainting for ssEM restoration

**DOI:** 10.1093/bioinformatics/btag329

**Published:** 2026-05-22

**Authors:** Zhenbang Zhang, Jingtong Feng, Hongjia Li, Haythem El-Messiry, Zhiqiang Xu, Renmin Han

**Affiliations:** Shenzhen Research Institute of Shandong University, Shenzhen, 518000, China; Research Center for Mathematics and Interdisciplinary Sciences, Frontiers Science Center for Nonlinear Expectations (Ministry of Education), Shandong University, Qingdao, 266237, China; Department of Machine Learning, Mohamed bin Zayed University of Artificial Intelligence, Abu Dhabi, 7909, United Arab Emirates; Research Center for Mathematics and Interdisciplinary Sciences, Frontiers Science Center for Nonlinear Expectations (Ministry of Education), Shandong University, Qingdao, 266237, China; College of Medical Information and Engineering, Ningxia Medical University, Ningxia, 750004, China; School of Medical Technology, Beijing Institute of Technology, Beijing, 100081, China; Canadian University Dubai, Dubai, 117781, United Arab Emirates; Department of Machine Learning, Mohamed bin Zayed University of Artificial Intelligence, Abu Dhabi, 7909, United Arab Emirates; Shenzhen Research Institute of Shandong University, Shenzhen, 518000, China; Research Center for Mathematics and Interdisciplinary Sciences, Frontiers Science Center for Nonlinear Expectations (Ministry of Education), Shandong University, Qingdao, 266237, China; College of Medical Information and Engineering, Ningxia Medical University, Ningxia, 750004, China

## Abstract

**Motivation:**

Serial section electron microscopy (ssEM) is essential for studying biological cell structures at nanometer resolution. However, supporting film folding (SFF) degradation frequently occurs during sample preparation, causing structural distortions and information loss that severely impair downstream analyses such as 3D reconstruction and neuron segmentation.

**Results:**

We propose RegInpaint, a novel recovery framework that jointly addresses deformation correction and missing-information restoration caused by SFF degradation. RegInpaint formulates SFF recovery as a joint problem of 3D elastic registration and image inpainting, providing a generalizable solution for ssEM restoration. Experiments on four EM datasets show that RegInpaint consistently outperforms existing methods in image restoration quality and significantly improves neuron segmentation accuracy.

**Availability and implementation:**

Source code is freely available at https://github.com/zhangzhenbang2021/RegInpaint.git.

## 1 Introduction

**Figure 1:**
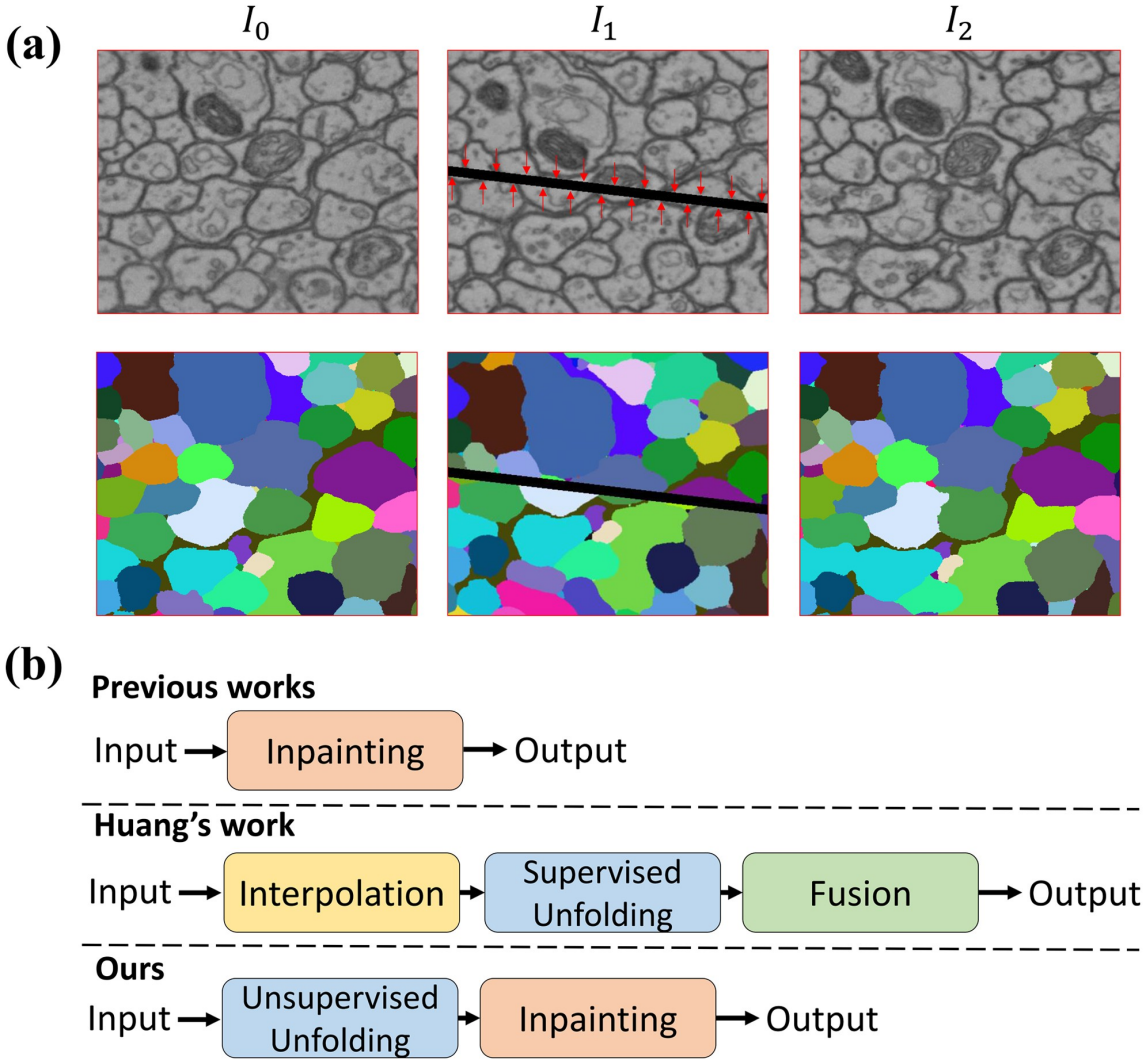
(a) Three consecutive ssEM images and their corresponding labels, where the middle one is degraded by Support Film Folding (SFF). (b) The workflow of previous methods and ours. The proposed method is carefully designed with two decoupled modules: unfolding and inpainting.

Serial section electron microscopy (ssEM) plays a crucial role in revealing synapse-level biological structures, greatly advancing research in neuronal morphology (Gour *et al.* 2021, Loomba *et al.* 2022) and connectomics (Yin *et al.* 2020, Xu *et al.* 2021), helping us gain a deeper understanding of the structural complexity of biological cell tissues (Yin *et al.* 2020, Ma and Wang 2023, Matsliah *et al.* 2024). For example, researchers successfully completed the first full electron microscope imaging of the adult fruit fly brain using ssEM technology (Zheng *et al.* 2018). The typical workflow of ssEM involves cutting biological samples into a series of microsections along the z-axis, followed by staining, digital imaging, 2D stitching (He *et al.* 2024), 3D registration (Popovych *et al.* 2024), and ultimately, 3D segmentation (Liu *et al.* 2024). However, during this process, slice damage and artifacts often arise due to manual operation errors and mechanical precision limitations (Popovych *et al.* 2024). Support film folding (SFF) degradation is one such common and challenging artifact (see Fig. 1a), which is not only widespread [affecting 3.2% of samples (Huang *et al.* 2020)] but also has a significant impact on image quality. This degradation phenomenon severely affects the accuracy of subsequent tasks such as image stitching (He *et al.* 2024), 3D registration (Xin *et al.* 2023, Popovych *et al.* 2024), and 3D segmentation (Liu *et al.* 2023a, b, Luo *et al.* 2024). For instance, in some neuron segmentation tasks (Li *et al.* 2019), images with SFF artifacts may interrupt the structure of multiple neurons, leading to incorrect reconstruction results.

The most prominent feature of SFF degradation is the loss of structural information in the image, which has led many studies (Zhang *et al.* 2020, Wang *et al.* 2021a, Cheng *et al.* 2024) to approach the problem from the perspective of image inpainting. For example, FlowInpaint (Cheng *et al.* 2024) proposes a two-stage reference generation strategy, first predicting intermediate state images, then using a GAN-based restoration network to integrate all reference information. However, these methods all overlook the structural deformations caused by SFF. Since SFF-degraded images typically exhibit large misalignments with neighboring images, this often leads to erroneous inpainting results. Huang *et al.* (2020) considered the impact of structural deformations and proposed a three-step recovery framework, consisting of interpolation, unfolding, and fusion. This framework generates interpolated images using neighboring images, and these are then used as references for the unfolding and fusion modules to address structural deformations and information loss. However, this method rely on optical flow from ground truth for supervised training in unfolding modules, which is difficult to obtain in practical applications.

However, many issues in the SFF degradation problem remain underexplored and unsolved. First is the issue of structural deformation. Previous methods have treated SFF degradation simply as an image inpainting problem (Wang *et al.* 2021a, Cheng *et al.* 2024) (see Fig. 1a), overlooking the impact of structural deformation on the quality of the generated images. A few works (Huang *et al.* 2020) have attempted to correct local deformations, but they require a large amount of manually annotated ground truth optical flow. Secondly, there is the issue of natural deformation between slices. Natural deformations cause morphological changes across adjacent slices, making it difficult to find the correct information to repair missing regions.

In this paper, we introduce RegInpaint, a novel ssEM image inpainting framework designed to simultaneously correct structural deformations and restore missing information caused by SFF degradation. We are the first to formulate SFF recovery as a joint 3D registration and image inpainting problem, carefully designing two decoupled modules: a registration (unfolding) module and an inpainting module (see Fig. 1b). The local region registration (LRR) module first aligns the SFF-degraded image with its neighboring slices, correcting structural distortions and ensuring consistency in the undamaged regions. Our method eliminates the need for ground truth optical flow. Following registration, the multi-scale deformable residual inpainting network (MDRIN) searches for repair information from adjacent slices to restore missing regions while ensuring axial continuity. We propose a multi-scale residual deformable alignment (MRDA) module, which can accurately locate repair information, overcoming morphological changes caused by natural deformations. To validate RegInpaint, we conduct extensive experiments on four EM datasets. Our results demonstrate that RegInpaint consistently outperforms existing approaches, significantly improving image quality and neuron segmentation accuracy across diverse datasets. Additionally, we show that our unsupervised Local Region Registration (LRR) module enhances the performance of existing inpainting methods, demonstrating its versatility and effectiveness as an independent component.

## 2 Materials and methods

### 2.1 Methods

#### 2.1.1 Problem setting and overview

**Figure 2:**
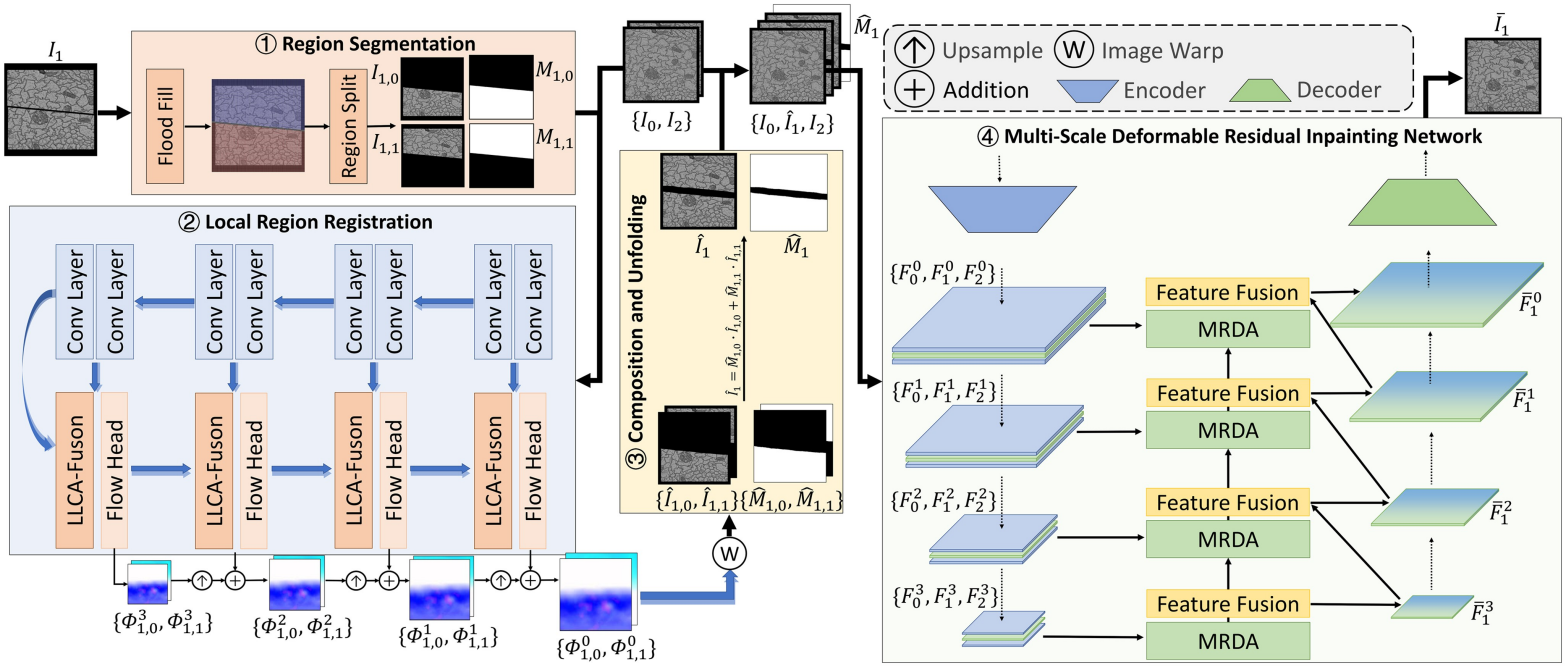
Overview of RegInpaint. It consists of two parts: unfolding and inpainting, which are specifically divided into: (1) flood fill-based region segmentation; (2) local correlation and channel attention-based local image registration module; (3) image synthesis and unfolding; (4) multi-scale deformable convolution-based image inpainting module.

Given a SFF degraded image $I_{1}$, and its neighboring images $\left\{I_{0},I_{2}\right\}$, along with the corresponding binary mask $M_{1}$, our goal is to synthesize accurate image details in the damaged regions and correct the image elastic distortion caused by SFF, thereby restoring axial structural consistency. Figure 2 illustrates the entire RegInpaint pipeline. First, we apply the flood-filling algorithm (Petrou and Petrou 2010) to segment $I_{1}$ and the corresponding mask $M_{1}$ into two parts: $\left\{I_{1,0},M_{1,0}\right\}$ and $\left\{I_{1,1},M_{1,1}\right\}$, based on the dark lines. Next, these segments, along with neighboring images $\left\{I_{0},I_{2}\right\}$, are then fed into the LRR module, which estimates the deformation fields $\left\{\Phi_{1,0},\Phi_{1,1}\right\}$. Third, we use $\left\{\Phi_{1,0},\Phi_{1,1}\right\}$ to warp $\left\{I_{1,0},I_{1,1}\right\}$ and $\left\{M_{1,0},M_{1,1}\right\}$, followed by a weighted synthesis to generate the unfolded image $\hat{I_{1}}$. Finally, $\hat{I_{1}}$ and $\left\{I_{0},I_{2}\right\}$ are processed by the MDRIN, which reconstructs the final restored image $\bar{I_{1}}$. We introduce the specific design of each component below. The following sections detail the design and functionality of each component.

#### 2.1.2 Region segmentation

The SFF-degraded image loses structural information in the dark line regions, introducing local distortions around them. Therefore, accurately correcting elastic deformation in these areas is the first step in restoration. However, the structural deformation typically occurs perpendicular to dark lines, with opposite deformation directions on either side (Huang *et al.* 2020). To address this, we first apply the flood-filling algorithm to segment the damaged image $I_{1}$ and its corresponding mask $M_{1}$ into two distinct regions, $\left\{I_{1,0},M_{1,0}\right\}$ and $\left\{I_{1,1},M_{1,1}\right\}$. These regions are then processed separately by the local registration module for accurate alignment and deformation correction.

#### 2.1.3 Local region registration

The two segmented parts, $\{I_{1,0},I_{1,1}\}\in R^{2B\times H\times W\times 1}$ and $\{M_{1,0},M_{1,1}\}\in R^{2B\times H\times W\times 1}$, along with the neighboring images $\{I_{0},I_{2}\}\in R^{B\times H\times W\times 1}$, are input into LRR module. LRR is biased toward correcting smooth SFF-induced physical distortion rather than forcing exact correspondence of all biological structures across adjacent sections. This is encouraged by two-sided similarity supervision from both neighboring slices, region-wise local unfolding around the dark line, and smoothness regularization on the deformation field. Consequently, isolated biological changes that appear in only one neighboring slice are less likely to induce stable deformation, whereas broad smooth mismatch patterns caused by SFF are consistently corrected. Nevertheless, very abrupt topology changes between consecutive sections remain challenging and cannot always be fully distinguished from artifact-induced displacement. Taking $I_{1,0}$ as an example, specifically, LRR first extracts multi-scale hierarchical features from $I_{0}$, $I_{1,0}$, and $I_{2}$, using an encoder, producing four levels of features: ${\left\{F_{0}^{i}\right\}}_{i=0}^{3}$, ${\left\{F_{1}^{i}\right\}}_{i=0}^{3}$, and ${\left\{F_{2}^{i}\right\}}_{i=0}^{3}$, respectively. Each set of features, $\left\{F_{0}^{i},F_{1}^{i},F_{2}^{i}\right\}$, is then processed by the Local Correlation and Channel Attention Fusion (LCCA) Module, which fuses motion information from adjacent slices. At each scale, the corresponding flow field $\Phi_{1,0}^{i}$ is computed, enabling a coarse-to-fine registration approach. Finally, we obtain the deformation fields $\Phi_{1,0}$ and $\Phi_{1,1}$ at the original resolution to unfold $I_{i}$.

**Local correlation and channel attention fusion.** The width of damaged regions can reach up to 80 pixels, indicating potential large displacements (Huang *et al.* 2020). Correlation, widely used in optical flow estimation (Teed and Deng 2020, Hui *et al.* 2021) and recently introduced in medical image registration (Meng *et al.* 2024), has demonstrated strong performance. We adopt local correlation to capture relationships between $F_{1,0}^{i}$ and its adjacent features, $F_{2}^{i}$ and $F_{0}^{i}$. The local correlation function $c_{t,s}^{l}(x,d)$ is defined as:


(1) $$c_{t,s}^{l}(x,d)=F_{t}^{l}{\left(x\right)}^{T}F_{s}^{l}(x+d),\;∥d{∥}_{\infty }\leq R,$$

where $x\in Z^{2}$ is the coordinate in the target feature map. $d\in Z^{2}$ is the displacement relative to the target coordinate. And *R* is the maximum displacement in any direction. Here, *l* denotes the level in the feature pyramid.

**Figure 3:**
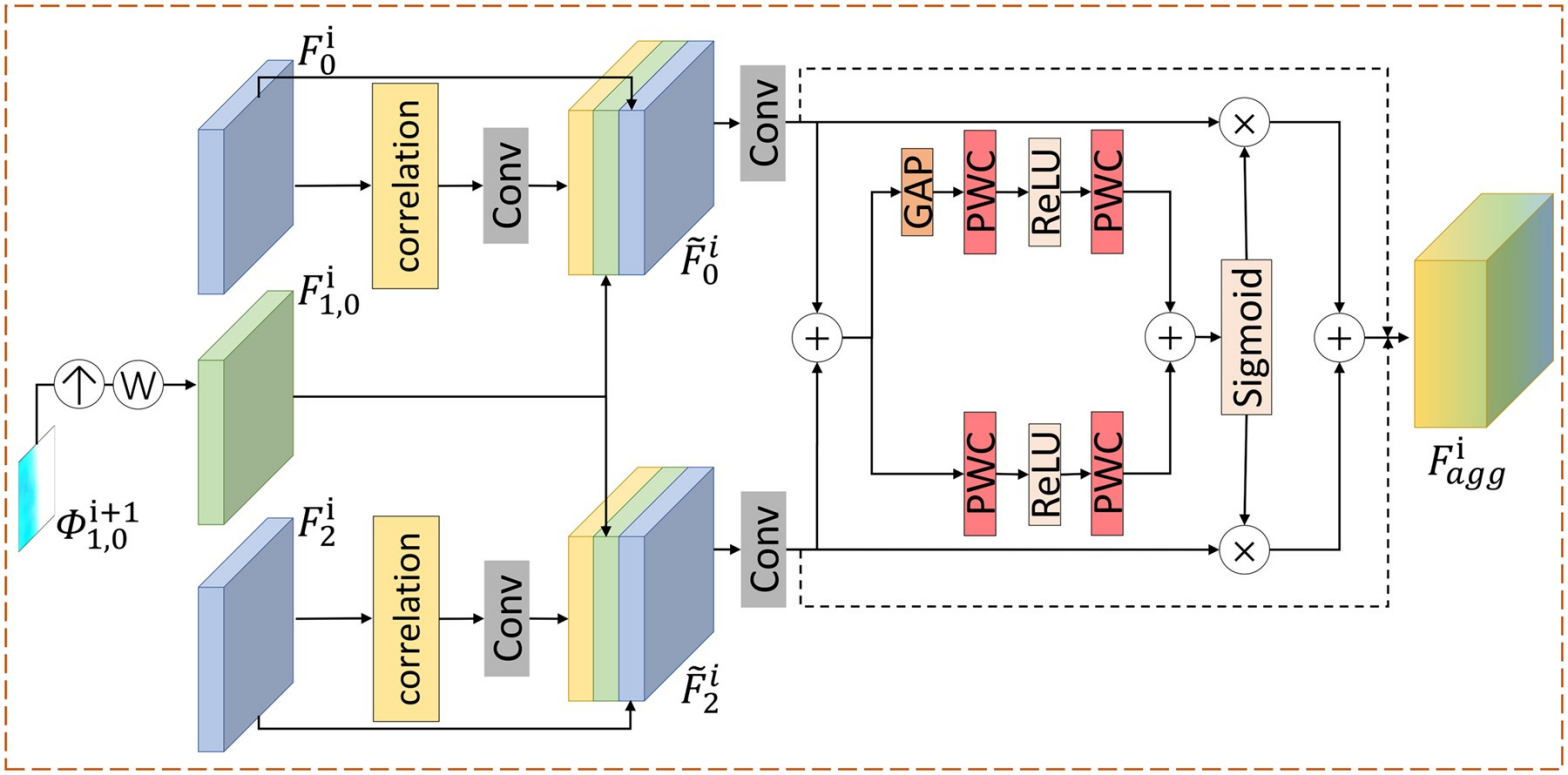
The local correlation and channel attention fusion module (LCCA). LCCA module is used to efficiently integrate displacement information and fuse feature from adjacent frames.

Unlike in medical image registration (Ma *et al.* 2024, Meng *et al.* 2024), where registration mainly concerns motion between moving and reference features, 3D EM image registration requires integrating motion features from both previous and next slices. Thus, effective feature fusion is critical (Hu *et al.* 2018, Dai *et al.* 2021). To achieve this, we introduce a global-local channel attention-based fusion module (see Fig. 3).

Specifically, given $F_{0}^{i}$ and $F_{2}^{i}$, along with $F_{1,0}^{i}$, we first compute local correlations and concatenate them with the original features along the channel dimension:


(2) $$\{\begin{array}{c} {\tilde{F}}_{0}^{i}=\text{Cat}[F_{0}^{i},F_{1,0}^{i},\text{Conv}(c_{0,\{1,0\}}^{i})],\\ {\tilde{F}}_{2}^{i}=\text{Cat}[F_{2}^{i},F_{1,0}^{i},\text{Conv}(c_{2,\{1,0\}}^{i})]. \end{array}$$

The concatenated features ${\tilde{F}}_{0}^{i}$ and ${\tilde{F}}_{2}^{i}$ are then processed by the feature fusion module. We use point-wise convolution (PWC) to emphasize both local and global features. The local feature attention L(X) is as follows:


(3) $$L(X)={\text{PWC}}_{2}(\delta ({\text{PWC}}_{1}(X))),$$

where PWC represents point-wise convolution and δ is the ReLU activation function. Similarly, global feature attention G(X) follows the same computation but first applies global average pooling (GAP) to *X*.

Finally, the channel attention fusion module computes the final aggregated feature:


(4) $$\{\begin{array}{c} \mathrm{Att}=\sigma (L({\tilde{F}}_{0}^{i}+{\tilde{F}}_{2}^{i})+G({\tilde{F}}_{0}^{i}+{\tilde{F}}_{2}^{i})),\\ F_{\mathrm{agg}}^{i}={\tilde{F}}_{0}^{i}+{\tilde{F}}_{2}^{i}+\mathrm{Att}⊗{\tilde{F}}_{0}^{i}+(1-\mathrm{Att})⊗{\tilde{F}}_{2}^{i}, \end{array}$$

where σ is the sigmoid function, ⊗ represents element-wise multiplication, and $F_{\mathrm{agg}}^{i}$ is the final aggregated feature.

#### 2.1.4 Composition and unfolding

After LRR, two local deformation fields $\Phi_{1,0}$ and $\Phi_{1,1}$, are generated. These fields are then used to warp the segmented image regions $\left\{I_{1,0},I_{1,1}\right\}$ and corresponding masks $\left\{M_{1,0},M_{1,1}\right\}$. Finally, a pixel-wise weighted fusion is performed to reconstruct the unfolded image $\hat{I_{1}}$:


(5) $$\{\begin{array}{c} {\hat{M}}_{1,0}=W(\Phi_{1,0},M_{1,0}),\\ {\hat{M}}_{1,1}=W(\Phi_{1,1},M_{1,1}),\\ \hat{I_{1}}={\hat{M}}_{1,0}⊗W(\Phi_{1,0},I_{1,0})+{\hat{M}}_{1,1}⊗W(\Phi_{1,1},I_{1,1}),\\ {\hat{M}}_{1}={\hat{M}}_{1,0}+{\hat{M}}_{1,1}, \end{array}$$

where W denotes the warping operation, and ⊗ represents element-wise multiplication.

#### 2.1.5 Deformation-aware residual inpainting

The unfolded image $\hat{I_{1}}$ is approximately aligned with the neighboring slices $I_{0}$ and $I_{2}$, but small nonlinear distortions and deformations remain. To refine the alignment, we propose the MDRIN, as illustrated in Fig. 2. Specifically, given $\left\{I_{0},I_{2}\right\}$, $\hat{I_{1}}$, and the mask ${\hat{M}}_{1}$, we first extract a five-level feature set ${\left\{F_{0}^{i},F_{1}^{i},F_{2}^{i}\right\}}_{i=0}^{4}$ using a multi-scale feature extractor (Fig. 2 shows only four levels due to space constraints). For each scale, the extracted features are processed by the MRDA module, which warps $F_{0}^{i}$ and $F_{2}^{i}$ to align them with $F_{1}^{i}$. We use two independent deformable convolutional networks (DCNs) to capture motion information more effectively. Additionally, we introduce residual offset refinement, where the deformable offsets from the previous scale are iteratively updated, improving alignment accuracy. Finally, a convolutional layer fuses the aligned features with $F_{1}^{i}$, and the result is combined with the refined feature ${\bar{F}}_{1}^{i+1}$ from the previous scale to produce the refined feature ${\bar{F}}_{1}^{i}$.

**Figure 4:**
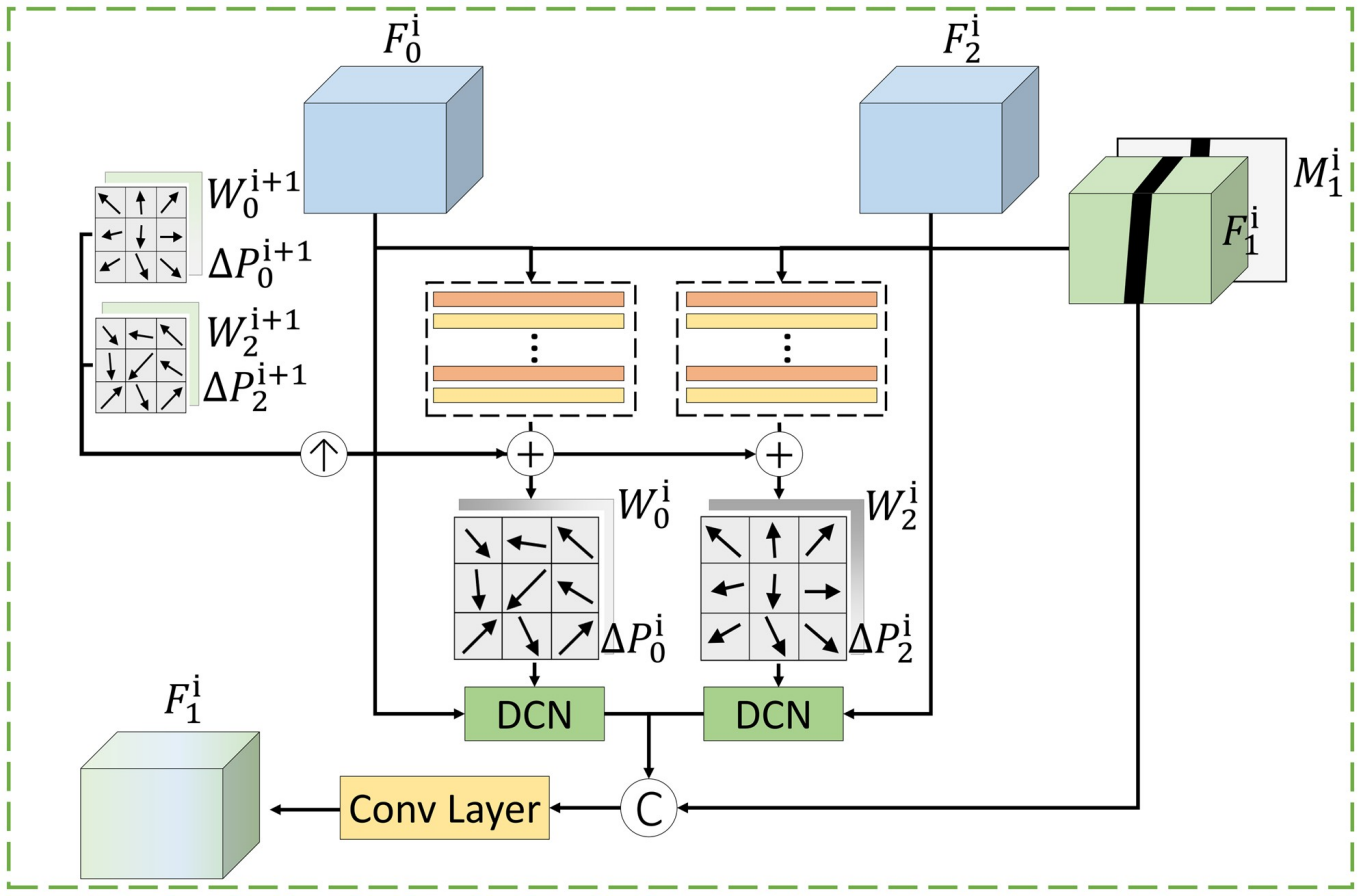
The MRDA module. We use the aligned features to fix the dark line.

**Multi-scale residual deformable alignment.** We propose the module to enhance the alignment of $F_{0}^{i},F_{2}^{i}$ using deformable convolutions (Wang *et al.* 2021b). MRDA leverages offset residuals from the previous layer to refine offset estimation, improving alignment accuracy (see Fig. 4). Compared with other attention-based fusion (Xiao *et al.* 2023a,b, 2026), MRDA has a distinct advantage because it corrects spatial misalignment rather than only reweighting features. Attention operates at fixed coordinates and therefore cannot recover membrane boundaries when their corresponding positions are shifted across slices. In contrast, deformable convolution samples neighboring features at adaptive locations determined by the learned offsets, allowing the model to follow thin, curved, and discontinuous membrane contours more accurately. The residual multi-scale offset refinement further separates large-scale displacement correction from fine-scale boundary refinement, which improves membrane continuity and consequently benefits affinity-based neuron segmentation.

Given the feature set $F_{0}^{i},F_{1}^{i},F_{2}^{i}$ at the current scale and the downsampled mask ${\hat{M}}_{1}^{i}$, we compute the offsets $\Delta P_{0}^{i}$ and $\Delta P_{2}^{i}$ for the previous and next slices using independent convolutional layers:


(6) $$\{\begin{array}{c} {}[W_{0}^{i},\Delta P_{0}^{i}]=\text{Conv}(F_{0}^{i},F_{1}^{i},M_{1}^{i}),\\ {}[W_{2}^{i},\Delta P_{2}^{i}]=\text{Conv}(F_{2}^{i},F_{1}^{i},M_{1}^{i}), \end{array}$$

where $\left\{W_{0}^{i},W_{2}^{i}\right\}$ are the weight mask and $\left\{\Delta P_{0}^{i},\Delta P_{2}^{i}\right\}$ are the offsets. At each pyramid level, the unfolded mask is downsampled to the same spatial resolution as the feature maps and used as an additional input channel for offset/weight prediction. Thus, the mask serves as a spatial reliability cue for deformable alignment, preventing corrupted dark-line regions in the current slice from misleading offset estimation. In regions with missing content, restoration is mainly guided by the aligned neighboring features, while the unfolded current-slice feature provides surrounding contextual information.

To improve alignment, we refine the offsets using residual connections from the previous layer:


(7) $$\{\begin{array}{c} {\hat{F}}_{0}^{i}=\text{DCN}(F_{0}^{i},W_{0}^{i},\Delta P_{0}^{i}+\Delta P_{0}^{i+1}),\\ {\hat{F}}_{2}^{i}=\text{DCN}(F_{2}^{i},W_{2}^{i},\Delta P_{2}^{i}+\Delta P_{2}^{i+1}). \end{array}$$

This iterative refinement enables more flexible sampling locations, improving semantic correspondences and progressively enhancing alignment from coarse to fine.

The warped features $F_{0}^{i}$ and $F_{2}^{i}$ along with $F_{1}^{i}$, are then passed through a 1×1 convolutional layer for adaptive fusion:


(8) $$F_{1}^{i}={\text{Conv}}_{1\times 1}({\hat{F}}_{0}^{i},F_{1}^{i},{\hat{F}}_{2}^{i}).$$

Finally, the refined feature $F_{1}^{i}$ is combined with the upsampled refined feature and ${\bar{F}}_{1}^{i+1}$ from the previous scale using the same fusion strategy as in Fig. 3, with the key difference being that the inputs are now $F_{1}^{i}$ and the upsampled ${\bar{F}}_{1}^{i+1}$. The final formulation is:


(9) $$\{\begin{array}{c} \mathrm{Att}=\sigma (L(F_{1}^{i}+{\bar{F}}_{1}^{i+1})+G(F_{1}^{i}+{\bar{F}}_{1}^{i+1})),\\ {\bar{F}}_{1}^{i}=F_{1}^{i}+{\bar{F}}_{1}^{i+1}+\mathrm{Att}⊗F_{1}^{i}+(1-\mathrm{Att})⊗{\bar{F}}_{1}^{i+1}, \end{array}$$

where σ represents the sigmoid function and ⊗ denotes element-wise multiplication.

#### 2.1.6 Training objectives

Previous works (Huang *et al.* 2020, Deng *et al.* 2022) have used ground truth optical flow to supervise unfolding and registration networks for SFF images. However, obtaining ground truth optical flow for EM images is challenging. To address this, we propose an unsupervised training strategy based on a similarity term and a regularization term.

We measure the average similarity between the unfolded image ${\hat{I}}_{1}$ (from Eq. 5) and its neighboring slices $\left\{I_{0},I_{2}\right\}$. The similarity loss is computed across multiple scales, and the total similarity loss $L_{\text{sim}}$ is defined as:


(10) $$L_{\text{sim}}=\frac{1}{L}\sum_{i=0}^{L}\frac{1}{2}\left(∥{\hat{I}}_{1}^{i}-I_{0}^{i}{∥}_{2}^{2}⊙{\hat{M}}_{1}^{i}+∥{\hat{I}}_{1}^{i}-I_{2}^{i}{∥}_{2}^{2}⊙{\hat{M}}_{1}^{i}\right),$$

where ${\hat{M}}_{1}^{i}$ is the downsampled unfolded valid-region mask at pyramid level *i* and ${\hat{I}}_{1}^{i}$ represents the output image at level *i*, $\left\{I_{0}^{i},I_{2}^{i}\right\}$ are the neighboring slices at the same level *i*, ${\hat{M}}_{1}^{i}$ is the downsampled mask at the level *i*, and *L* denotes the number of pyramid levels.

To ensure that the deformation fields $\left\{\Phi_{1,0}^{i},\Phi_{1,1}^{i}\right\}$ remain smooth and physically plausible, we introduce a regularization loss:


(11) $$L_{\text{reg}}=\frac{1}{L}\sum_{i=0}^{L}\left(||▽\Phi_{1,0}^{i}|{|}_{2}^{2}+||▽\Phi_{1,1}^{i}|{|}_{2}^{2}\right).$$

The final total loss function combines both terms:


(12) $$L=L_{\text{sim}}+\lambda L_{\text{reg}},$$

where λ denotes the regularization weight parameter.

We optimize the inpainting network using three loss functions: a reconstruction loss and two adversarial losses. The pixel-wise L1 loss measures the difference between the reconstructed image $\bar{I_{1}}$ and the ground truth $I_{1}^{\text{gt}}$:


(13) $$L_{\text{rec}}=||I_{1}^{\text{gt}}-\bar{I_{1}}|{|}_{1}.$$

#### 2.1.7 Network architecture

**Local region registration.** The LRR adopts an encoder–decoder architecture. The encoder contains four convolutional layers with channel numbers of 16, 32, 64, and 128. Each layer includes two convolutions: a 3×3 convolution followed by a 7×7 convolution to enlarge the receptive field, following LKUnet (Jia *et al.* 2022). Given an input image of size (512, 512), the feature map resolutions are progressively reduced to (256, 256), (128, 128), (64, 64), and (32, 32).

The decoder consists of four stages, each composed of a Local Correlation and Channel Attention Fusion Module (LCCA) and a Flow Head. Each Flow Head includes two 3×3 convolutions to predict an optical flow field at the corresponding scale. The predicted flow is upsampled and used to warp features at the next level via a spatial transformer network (STN) (Jaderberg *et al.* 2015). In the final stage, a learnable upsampling layer is introduced to generate full-resolution optical flow. Following RAFT (Teed and Deng 2020), the network first predicts half-resolution flow and then upsamples it to full resolution using a convex combination of neighboring flow vectors in a 3×3 window, with weights predicted by two convolution layers.

**Multi-scale deformable residual inpainting.** In the MDRIN, the encoder consists of five convolutional layers, with the output channel sizes being 8, 16, 32, 64, and 128, respectively. We adopt the same encoder architecture as in the LRR network, where each layer contains two convolution operations: the first convolution uses a 3×3 kernel, and the second convolution uses a larger 7×7 kernel to increase the receptive field of the network. The input image size is (512, 512), and after each convolutional layer, the output feature map size gradually decreases to (512, 512), (256, 256), (128, 128), (64, 64), and (32, 32).

The decoder consists of five layers, each equipped with a MRDA module. In the MRDA module, we align the features of adjacent slices using two independent deformable convolutions, which help capture more accurate repair information. The deformable convolutions have a kernel size of 3×3 and a group size of 8. These convolutions are designed to learn different deformation patterns, improving the alignment accuracy of structural information in the image. For each output feature from the repair process, we apply a transposed convolution layer for upsampling. The upsampled features are then passed to the MRDA module in the next layer, enabling fine-to-coarse inpainting.

### 2.2 Datasets

The CREMI dataset (Funke *et al.* 2016) consists of neuronal slices from the adult *Drosophila melanogaster* brain and includes three image stacks (CREMIA, CREMIB, and CREMIC), each containing 125 consecutive sections with a spatial resolution of 1250×1250 pixels. The data were acquired using serial section transmission electron microscopy (ssTEM) with a voxel spacing of 4×4×40 nm. The AC34 dataset comprises two labeled subvolumes cropped from the mouse somatosensory cortex dataset Kasthuri15 (Kasthuri *et al.* 2015), acquired at a resolution of 3×3×29 nm. The EPFL dataset (Lucchi *et al.* 2013) is derived from a 5 × 5 × 5 μm region of the CA1 hippocampus in the mouse brain, corresponding to a volume of 1065×2048×1536 pixels with an isotropic voxel resolution of approximately 5×5×5 nm.

Following the experimental protocol of Huang *et al.* (2020), SFF degradation was applied to the above datasets to construct training and evaluation samples. For CREMI, we manually annotated 4631 anchor locations in unlabeled volumes and extracted 4631 training samples, each consisting of three consecutive slices with an original patch size of 1024×1024 pixels. SFF degradation was applied to the middle slice of each triplet. The patches were then enhanced using contrast-limited adaptive histogram equalization (CLAHE) and resized to 512×512 pixels. For the EPFL and AC34 datasets, we cropped 5000 training samples per dataset, each with an original size of 512×512 pixels, and applied the same preprocessing and degradation procedures.

For quantitative evaluation, we extracted 200 test samples from each CREMI subset, 200 samples from the EPFL dataset, and 200 samples from each AC34 subset. All test samples were subjected to the same SFF degradation process as the training data.

In addition, we evaluated our method on the FAFB dataset (Zheng *et al.* 2018), which represents the first complete electron microscopy volume of an adult *Drosophila* brain. The dataset was imaged at a lateral resolution of 4×4 nm with a section thickness of 40 nm. A subset of slices exhibiting clear SFF degradation was manually selected and used exclusively for evaluation.

## 3 Results

### 3.1 Experimental settings

RegInpaint is conducted on an NVIDIA A100 GPU with 40 GB of memory. For the LRR network, we use a learning rate of 0.0001, a batch size of 12, and set the regularization parameter θ to 0.005. For the inpainting network, we adopt a learning rate of 0.0001 and a batch size of 8. For LRR network, we use a learning rate of 0.0001, a batch size of 12, training for 500 epochs. For MDRIN, we adopt a learning rate of 0.0001 and a batch size of 8, training for 500 epochs. Both modules are optimized using the Adam optimizer with $\beta_{1}$ = 0.5 and $\beta_{2}$ = 0.999. To demonstrate that RegInpaint can generate realistic and correct texture structures, we tested its performance on downstream segmentation tasks. We used the state-of-the-art neuron cell segmentation algorithm CAD (Liu *et al.* 2024) and used its pre-trained model, which was trained on the CREMI dataset. Specifically, we input three consecutive SFF-degraded EM images and used CAD to estimate the corresponding affinity maps. Finally, we applied the Waterz algorithm (Funke *et al.* 2019) for post-processing to obtain the final segmentation results. For metrics, we used PSNR, SSIM, and FID (Heusel *et al.* 2017) for quantitative comparison. PSNR and SSIM assess pixel-level similarity, while FID compares feature distributions using high-level perceptual features from InceptionV3 (Szegedy *et al.* 2016).

Synthetic SFF degradation was generated in three steps. First, two random endpoints on different image boundaries were sampled to define a straight dark line, together with its visible width $w_{1}$ and the width of the actually affected region $w_{2}$. Second, a folded optical flow field was synthesized with direction perpendicular to the dark line and amplitude linearly decaying with radial distance to the line: $k_{f}=-1/k_{l}$, $d(i,j)=\left|\frac{k_{l}i-j+b_{l}}{\sqrt{k_{l}^{2}+1}}\right|$, $A(i,j)=\alpha d(i,j)+b_{f}$, and $F(i,j,:)=(A(i,j)\;\text{cos}(\arctan(k_{f})),A(i,j)\;\text{sin}(\arctan(k_{f})))$. Third, the artifact-free image was warped by this flow field and a black line of width $w_{1}$ was overlaid to form the final degraded image. The binary mask was obtained directly from the simulated dark-line region and therefore was noise-free in the synthetic setting.

### 3.2 Comparison with existing methods

We compared the proposed RegInpaint framework with four representative inpainting methods, including two approaches specifically designed for EM image restoration [SFF (Huang *et al.* 2020) and FlowInpaint (Cheng *et al.* 2024)] and two general video inpainting methods [E2FGVI (Li *et al.* 2022) and ProPainter (Zhou *et al.* 2023)]. In addition, we evaluated the effect of integrating the proposed unsupervised LRR module with these methods.

Quantitative results are summarized in Table 1. RegInpaint consistently achieves the best performance across most evaluation metrics, indicating its effectiveness in restoring structural consistency and image quality in SFF-degraded ssEM slices. Moreover, incorporating the LRR module leads to consistent performance improvements for multiple baseline methods, demonstrating that LRR effectively corrects SFF-induced structural distortions and can function as a general pre-alignment module. Although RegInpaint does not achieve the highest PSNR on every subset, PSNR is not the sole indicator of restoration quality in ssEM. Because PSNR measures pixel-wise error, it may favor over-smoothed predictions that reduce intensity differences but weaken thin membrane boundaries and structural continuity. In contrast, for connectomics applications, preserving biologically meaningful topology and axial consistency is more important. This is why we additionally report FID and downstream segmentation metrics, which better reflect structural realism and biological utility.

**Table 1:** Quantitative results compared with SFF (Huang *et al.* 2020), FlowInpaint (Cheng *et al.* 2024), E2FGVI (Li *et al.* 2022), and ProPainter (Zhou *et al.* 2023) on three datasets.

		SFF	FlowInpaint	E2FGVI	ProPainter	FlowInpaint^a^	E2FGVI^a^	ProPainter^a^	RegInpaint
CREMI A	PSNR	23.66	22.84	22.98	22.77	24.93↑2.09	24.45↑1.47	24.54↑1.78	**24.74**
	SSIM	0.77	0.65	0.67	0.66	0.79↑0.14	0.79↑0.11	0.80↑0.14	**0.81**
	FID	25.43	38.54	24.14	23.05	20.92↓17.62	22.11↓2.03	20.51↓2.55	**13.00**
CREMI B	PSNR	23.56	22.77	22.96	22.72	**24.95** ↑2.18	24.45↑1.50	24.46↑1.74	24.89
	SSIM	0.74	0.60	0.62	0.60	0.77↑0.16	0.76↑0.13	0.77↑0.16	**0.78**
	FID	40.43	38.08	27.34	27.25	21.93↓16.15	24.54↓2.79	23.95↓3.30	**14.27**
CREMI C	PSNR	22.77	21.82	22.08	21.82	**24.07** ↑2.26	23.61↑1.53	23.54↑1.72	24.02
	SSIM	0.72	0.58	0.60	0.59	0.76↑0.18	0.75↑0.15	0.76↑0.17	**0.77**
	FID	29.30	41.53	29.81	29.35	23.28↓18.26	24.17↓5.64	20.53↓8.82	**13.43**
AC3	PSNR	16.22	16.53	15.19	15.17	16.73↑0.20	16.77↑1.58	**16.97** ↑1.81	16.72
	SSIM	0.64	0.62	0.49	0.47	0.65↑0.03	0.65↑0.17	0.67↑0.20	**0.67**
	FID	89.99	96.69	87.23	84.46	72.08↓24.61	66.71↓20.51	66.14↓18.32	**61.50**
AC4	PSNR	15.67	15.93	14.68	14.67	16.21↑0.27	16.23↑1.55	**16.45** ↑1.77	16.12
	SSIM	0.61	0.59	0.45	0.44	0.63↑0.04	0.63↑0.17	0.64↑0.20	**0.64**
	FID	99.24	105.60	91.79	86.54	81.25↓24.35	76.61↓15.19	75.46↓11.08	**65.87**
EPFL	PSNR	20.75	19.27	19.92	20.64	20.19↑0.92	21.12↑1.20	20.92↑0.28	**21.67**
	SSIM	0.83	0.72	0.75	0.79	0.80↑0.07	0.85↑0.09	0.83↑0.04	**0.88**
	FID	55.63	52.09	50.31	56.66	49.53↓2.55	47.56↓2.75	48.80↓7.86	**35.20**

a Means the incorporation of the LRR module.

Bold values indicate the best performance.

**Figure 5:**
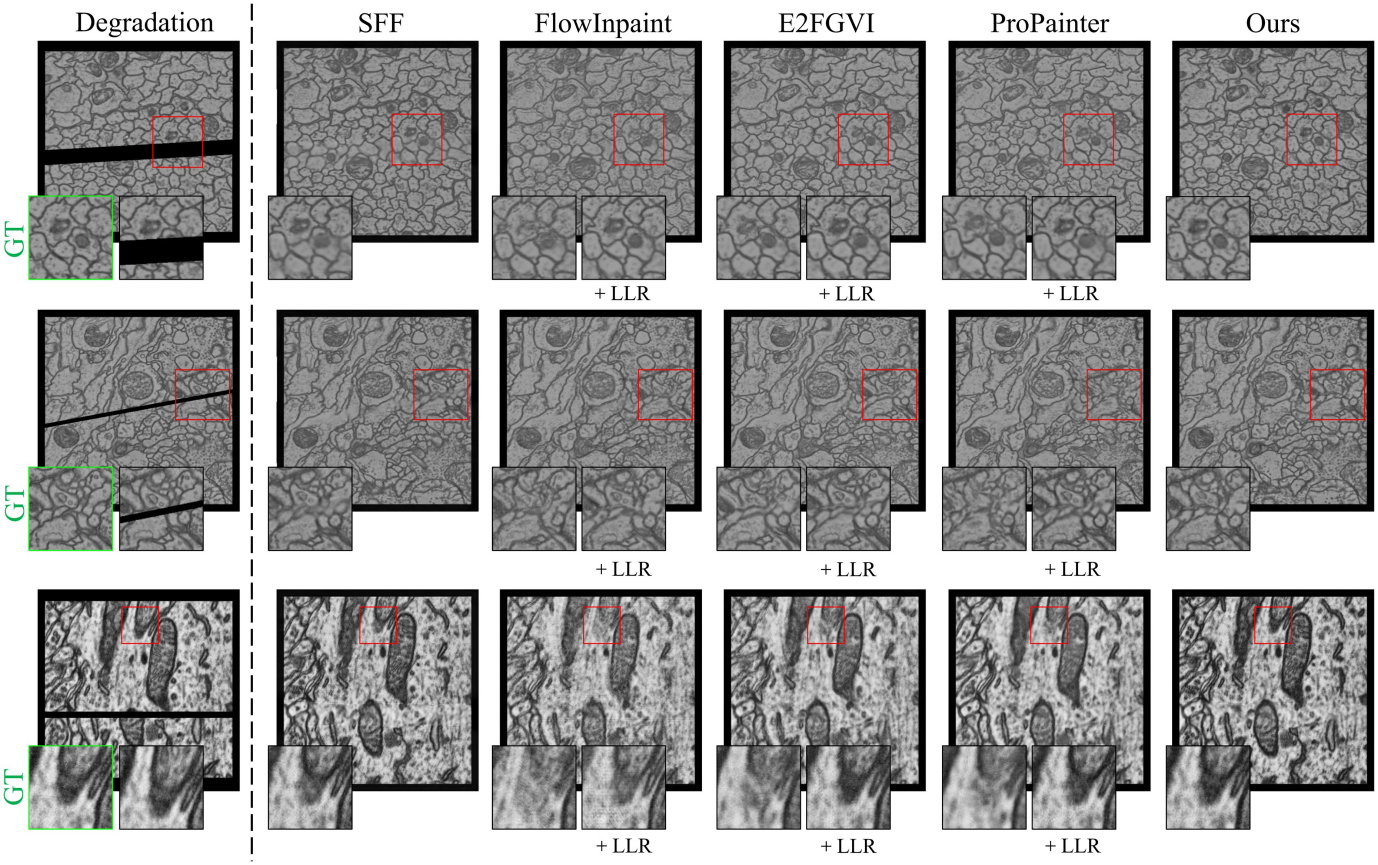
Qualitative results compared with SFF (Huang *et al*. 2020), FlowInpaint (Cheng *et al*. 2024), E2FGVI (Li *et al*. 2022), and ProPainter (Zhou *et al*. 2023). RegInpaint demonstrates superiority in generating complete and faithful textures, resulting in biologically plausible structures.

**Figure 6:**
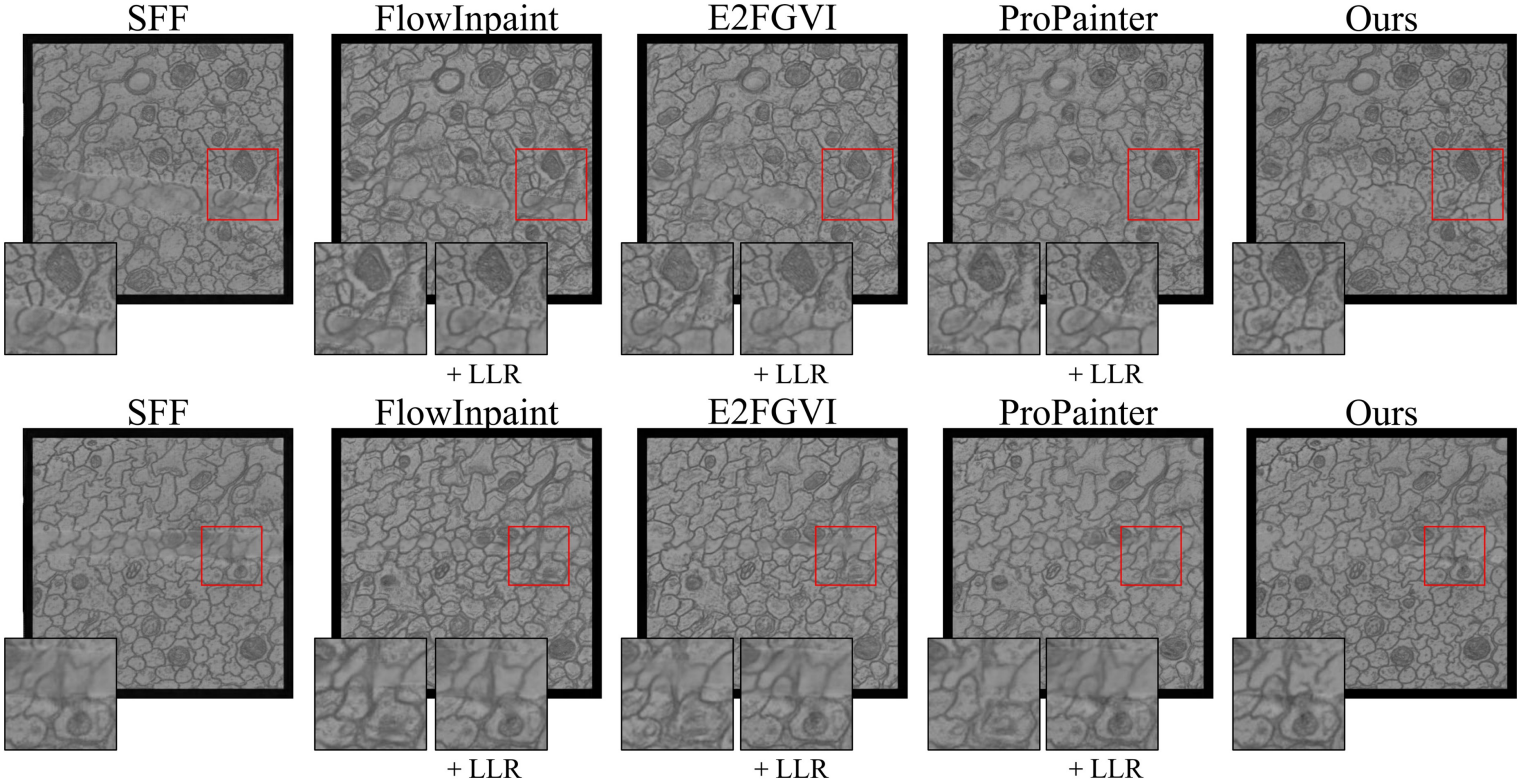
Qualitative results on FAFB dataset. RegInpaint demonstrates superiority in generating complete and faithful textures, resulting in biologically plausible structures.

Figures 5 and 6 present representative visual comparisons between RegInpaint and competing methods. Existing approaches struggle to simultaneously address structural deformation and information loss caused by SFF degradation. For instance, FlowInpaint often fails to recover continuous neuronal membranes, whereas the introduction of LRR improves alignment and yields more coherent restorations.

In addition, the last row of Fig. 5 illustrates restoration results in non-dark-line regions under large displacement deformation. While comparison methods exhibit artifacts or inconsistencies in these regions, RegInpaint produces more coherent textures and preserves structural continuity, highlighting its robustness to large deformations.

### 3.3 Impact on downstream neuron segmentation

SFF degradation can severely disrupt neuronal structures, leading to inaccurate segmentation and reconstruction results (Li *et al.* 2019). To assess whether RegInpaint improves downstream analysis, we conducted neuron segmentation experiments using CAD (Liu *et al.* 2024) to generate affinity maps and segmentations. Segmentation accuracy was evaluated using variation of information (VOI) (Meilă 2003) and adapted Rand error (ARAND) (Unnikrishnan *et al.* 2007).

**Figure 7:**
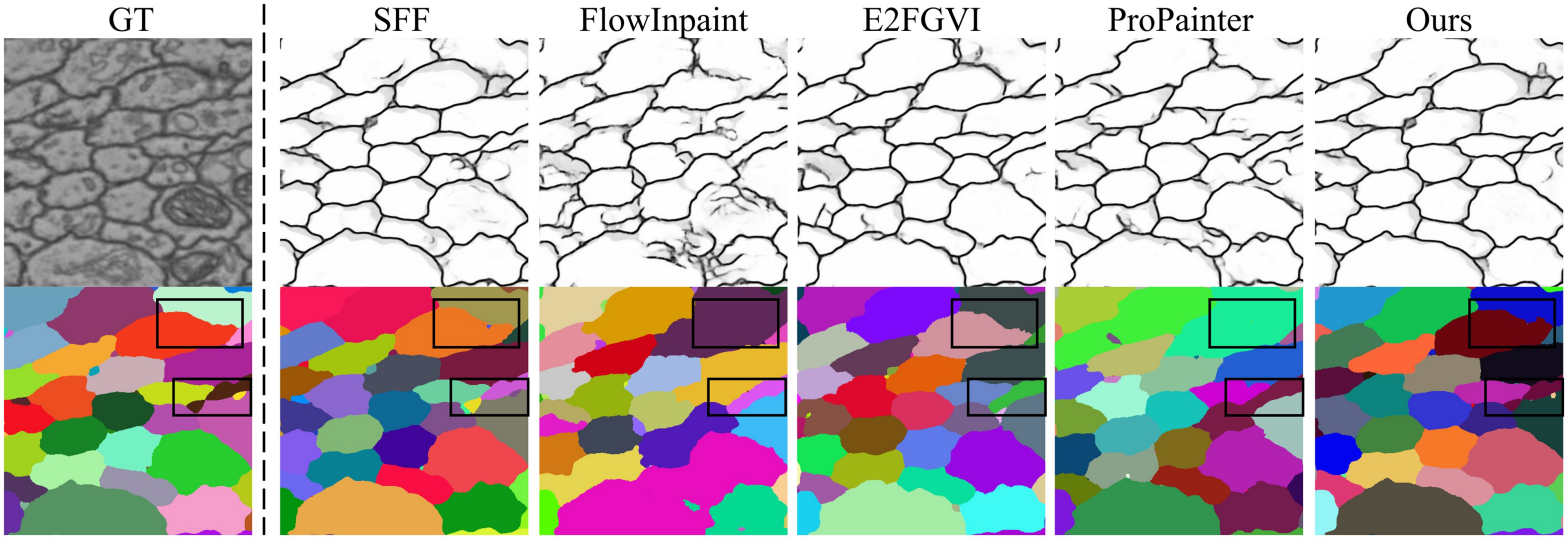
Qualitative neuron segmentation results compared with SFF (Huang *et al*. 2020), FlowInpaint (Cheng *et al*. 2024), E2FGVI (Li *et al*. 2022), and ProPainter (Zhou *et al*. 2023).

Quantitative results are reported in Table 2. RegInpaint achieves lower VOI and ARAND values than competing methods, indicating improved segmentation accuracy. Visual comparisons in Fig. 7 further show that RegInpaint produces more faithful neuronal structures, resulting in more accurate affinity maps and fewer split and merge errors. These results demonstrate that improved image restoration translates into tangible benefits for downstream biological analysis.

**Table 2:** Inpainting results on neuron segmentation task.

	CREMI A		CREMI B		CREMI C	
	VOI↓	ARAND↓	VOI↓	ARAND↓	VOI↓	ARAND↓
SFF	0.689	0.142	1.470	0.354	2.214	0.490
FlowInpaint	0.881	0.179	1.851	0.387	2.567	0.531
E2FGVI	0.795	0.163	1.730	0.375	2.411	0.510
ProPainter	0.840	0.171	1.741	0.369	2.513	0.522
Ours	**0.577**	**0.115**	**1.365**	**0.322**	**1.991**	**0.448**

Bold values indicate the best performance.

### 3.4 Ablation studies

We conducted ablation studies on the CREMI dataset to examine the contribution of key components in the proposed framework.

#### 3.4.1 Effect of the LCCA module

The local correlation and channel attention (LCCA) module is designed to integrate displacement information from adjacent slices. Its effect was evaluated by measuring PSNR and SSIM in non-dark-line regions between unfolded images and ground truth. As shown in Table 3, removing the LCCA module leads to a noticeable performance drop. Incorporating local correlation improves accuracy by capturing inter-slice motion, while channel attention further enhances performance by adaptively fusing features from neighboring slices.

**Table 3:** Ablation study on LCCA module.

Case	CREMI A		CREMI B		CREMI C	
	PSNR	SSIM	PSNR	SSIM	PSNR	SSIM
w/o LCCA	23.53	0.78	24.02	0.79	23.39	0.78
+ Corrlation	24.42	0.83	25.56	0.81	24.77	0.80
+ CA	24.10	0.80	25.44	0.81	24.71	0.79
Full Module	25.78	0.85	26.03	0.83	25.09	0.83

#### 3.4.2 Effect of the MRDA module

The MRDA module aims to precisely align neighboring features and facilitate texture recovery in dark-line regions. Results in Table 4 show that removing MRDA significantly degrades inpainting performance, reflecting the difficulty of accurately locating texture information without deformable alignment. Introducing deformable convolutions improves restoration quality, and further gains are achieved by incorporating residual offset refinement, which enables more accurate multi-scale alignment and improves structural consistency.

**Table 4:** Ablation study on MRDA module.

Case	CREMI A		CREMI B		CREMI C	
	PSNR	SSIM	PSNR	SSIM	PSNR	SSIM
w/o MRDA	23.13	0.75	23.81	0.74	23.09	0.73
+ DCNs	24.02	0.78	24.55	0.77	23.87	0.75
+ Residuals	24.74	0.81	24.89	0.78	24.02	0.77

#### 3.4.3 Effect of multi-scale residual deformable alignment

To further isolate the contribution of the inpainting-stage alignment module, we compared three variants: (i) *Standard Conv Alignment*, where deformable convolution is replaced by standard convolution; (ii) *DCN w/o Residual Offsets*, where deformable convolution is retained but cross-scale residual offset refinement is removed; and (iii) the full *MRDA*. As shown in Table 5, replacing deformable alignment with standard convolution leads to inferior performance, indicating that fixed-location sampling is insufficient to compensate for local non-rigid misalignment across adjacent slices. Introducing deformable convolution improves restoration quality by adaptively shifting the sampling positions. Further incorporating residual offset refinement yields the best performance, showing that propagating coarse alignment cues to finer scales improves structural continuity and fine-scale recovery.

**Table 5:** Ablation study of the alignment module on Cremi A dataset.

Method	PSNR ↑	SSIM ↑	FID ↓
Standard conv alignment	23.01	0.75	14.52
DCN w/o residual offsets	23.77	0.78	13.85
Full MRDA	24.74	0.81	13.00

#### 3.4.4 Sensitivity to the local-correlation radius

In all experiments, the same local-correlation radius *R* was used across all datasets, including both *Drosophila* and mammalian tissues. We further evaluated the sensitivity of the method to *R* by varying it while keeping all other settings fixed. As shown in Table 6, increasing *R* beyond the selected value yields only marginal performance improvement. This is because, in the local correlation formulation


(14) $$c_{t,s}^{l}(x,d)=F_{t}^{l}{\left(x\right)}^{T}F_{s}^{l}(x+d),\;∥d{∥}_{\infty }\leq R,$$

each spatial location *x* must evaluate all candidate displacements within a square search window of size (2R+1)×(2R+1). Therefore, the size of the correlation volume grows proportionally to


(15) $${\left(2R+1\right)}^{2},$$

which means that both computation and memory increase quadratically with *R*. In practice, once the dominant local displacement is covered, a much larger *R* is not cost-effective.

**Table 6:** Sensitivity analysis of the correlation radius *R* on Cremi A dataset.

*R*	PSNR ↑	SSIM ↑	FID ↓
1	24.74	0.81	13.00
2	24.89	0.83	13.71
4	24.96	0.83	13.90

#### 3.4.5 Effect of deformable group count

We also analyzed the effect of the deformable group count *G* in MRDA. Let the deformable kernel size be K×K. For a feature map of size $H_{i}\times W_{i}$, the offset and modulation branches predict


(16) $$\Delta P^{i}\in R^{H_{i}\times W_{i}\times 2K^{2}G},\;W^{i}\in R^{H_{i}\times W_{i}\times K^{2}G}.$$

Hence, the number of predicted channels is $3K^{2}G$, which implies that the offset/mask prediction cost and memory both grow linearly with *G*. If the offset branch is implemented by a convolution from a feature map with $C_{i}$ channels, its complexity is approximately


(17) $$O\left(H_{i}W_{i}C_{i}\cdot 3K^{2}G\right),$$

and the memory required for offsets and masks is


(18) $$O\left(H_{i}W_{i}\cdot 3K^{2}G\right).$$

Thus, increasing *G* provides more flexible group-wise sampling patterns for aligning thin and irregular structures, but also increases computation and memory nearly proportionally. As shown in Table 7, increasing the group count from 4 to 8 improves restoration quality, while increasing it further to 12 brings only marginal gain relative to the additional cost. Therefore, we use G=8 as the best trade-off between accuracy and efficiency.

**Table 7:** Ablation study of the deformable group count *G* on Cremi A dataset.

*G*	PSNR ↑	SSIM ↑	FID ↓	Time (ms) ↓
4	20.03	0.76	17.52	55
8	24.74	0.81	13.00	89
12	25.07	0.84	13.83	127

#### 3.4.6 Runtime analysis of residual offset refinement

We further evaluated whether the residual offset refinement in MRDA substantially increases inference time. In a single-scale deformable alignment, the aligned features are obtained using only the offsets predicted at the current scale:


(19) $${\hat{F}}_{0}^{i}=\text{DCN}(F_{0}^{i},W_{0}^{i},\Delta P_{0}^{i}),\;{\hat{F}}_{2}^{i}=\text{DCN}(F_{2}^{i},W_{2}^{i},\Delta P_{2}^{i}).$$

By contrast, MRDA refines the current-scale offsets by incorporating the propagated offsets from the coarser scale:


(20) $${\hat{F}}_{0}^{i}=\text{DCN}(F_{0}^{i},W_{0}^{i},\Delta P_{0}^{i}+\Delta P_{0}^{i+1}),\;{\hat{F}}_{2}^{i}=\text{DCN}(F_{2}^{i},W_{2}^{i},\Delta P_{2}^{i}+\Delta P_{2}^{i+1}).$$

The additional operation introduced by residual refinement is therefore only the cross-scale offset propagation and element-wise summation,


(21) $$\Delta P_{\text{total}}^{i}=\Delta P^{i}+\Delta P^{i+1},$$

rather than an iterative test-time optimization loop. As a result, its extra cost is much smaller than the dominant cost of deformable convolution itself. Table 8 confirms that the major runtime gap lies between single-scale and multi-scale deformable alignment, while the overhead of residual offset refinement is small.

**Table 8:** Inference-time comparison of different alignment strategies in the inpainting stage on Cremi A dataset.

Method	Inference time (ms) ↓
Single-scale alignment	47
MRDA w/o residual offsets	61
Full MRDA	64

## 4 Discussion

This study presents RegInpaint, a unified framework for restoring serial section electron microscopy (ssEM) images affected by support film folding (SFF) degradation. Accurate restoration of ssEM data is essential for downstream analyses in connectomics, as SFF-induced deformation and information loss can severely disrupt neuronal morphology and compromise 3D reconstruction and segmentation. Unlike previous approaches that primarily treat SFF degradation as an image inpainting problem, RegInpaint explicitly addresses both structural deformation and missing-content recovery within a single framework. A key strength of the proposed method lies in its formulation of SFF recovery as a joint problem of deformation correction and image inpainting. By introducing a registration-guided restoration strategy, RegInpaint first corrects elastic distortions using neighboring slices and then performs deformation-aware inpainting to restore missing regions. This design allows the method to preserve axial structural consistency while avoiding the over-smoothing or misalignment often observed in inpainting-only approaches. The experimental results demonstrate that this joint formulation leads to consistent improvements in image quality and structural continuity across multiple datasets and imaging conditions. Another important aspect of RegInpaint is its fully unsupervised deformation correction strategy. By optimizing feature-level similarity across adjacent slices and enforcing smoothness constraints on deformation fields, RegInpaint effectively corrects SFF-induced distortions without requiring explicit deformation annotations. Beyond image restoration, RegInpaint provides tangible benefits for downstream biological analysis. Improvements in neuron segmentation accuracy observed in our experiments indicate that restoring structural continuity at the image level directly translates into more reliable affinity estimation and segmentation outcomes. This observation underscores the importance of addressing SFF degradation not only as a visual restoration problem but also as a critical preprocessing step for connectomics pipelines.

## Supplementary Material

btag329_Supplementary_Data

## Data Availability

The four datasets used in this study are all publicly available. The CREMI dataset can be accessed at http://cremi.org/. The EPFL dataset is available at https://www.epfl.ch/labs/cvlab/data/data-em/. The AC3 and AC4 datasets can be downloaded from https://software.rc.fas.harvard.edu/lichtman/vast/AC3AC4Package.zip. The FAFB dataset is available at https://temca2data.org/.
