# Supplementary material for: 3D registration-guided deformable residual inpainting for ssEM restoration
Source: Bioinformatics. 2026 May 22;42(6):btag329. doi: 10.1093/bioinformatics/btag329 (PMC13245855; doi:10.1093/bioinformatics/btag329)
Supplement: btag329_Supplementary_Data [file btag329_supplementary_data.pdf]

Supplementary materials for “3D Registration-Guided Deformable Residual Inpainting for ssEM Restoration”

1. More Experiments

1.1. More Qualitative Results

We provide more visualization results on three datasets: CREMI, AC34, and EPFL. As shown in Figures 1 to 5, compared with other methods, RegInpaint can produce more faithful texture and structural information, as well as more coherent content in the damaged areas. Furthermore, we present additional segmentation results in Figure 6, where it can be observed that RegInpaint is able to restore the correct structural information, thereby improving the accuracy of downstream segmentation tasks.

1.2. Explainable Visualization Study

We present more explainable visualization results in Figure 7. Figure 7(b) shows the deformation field generated by the local region registration (LLR) module. As seen from the figure, the deformation fields on both sides of the dark line are nearly oppositely directed, which validates the necessity of using the flood-filling method for region segmentation: it effectively reduces the conflict between opposing deformation field directions and smoothness regularization. Figure 7(d) and (e) show the comparison of feature maps before and after restoration. The restored feature map successfully recovers missing structural information and significantly enhances the features in the dark line regions.

2. Limitation and Future Work

Figure 8 shows two failure cases. When large motion occurs across slices, our method struggles to capture the correct restoration information, resulting in artifacts and incorrect textures in the dark line regions. This indicates that such cases still pose a challenge for EM image restoration. Therefore, exploring more approaches in the future to preserve edge texture in the generated regions is a foreseeable direction.

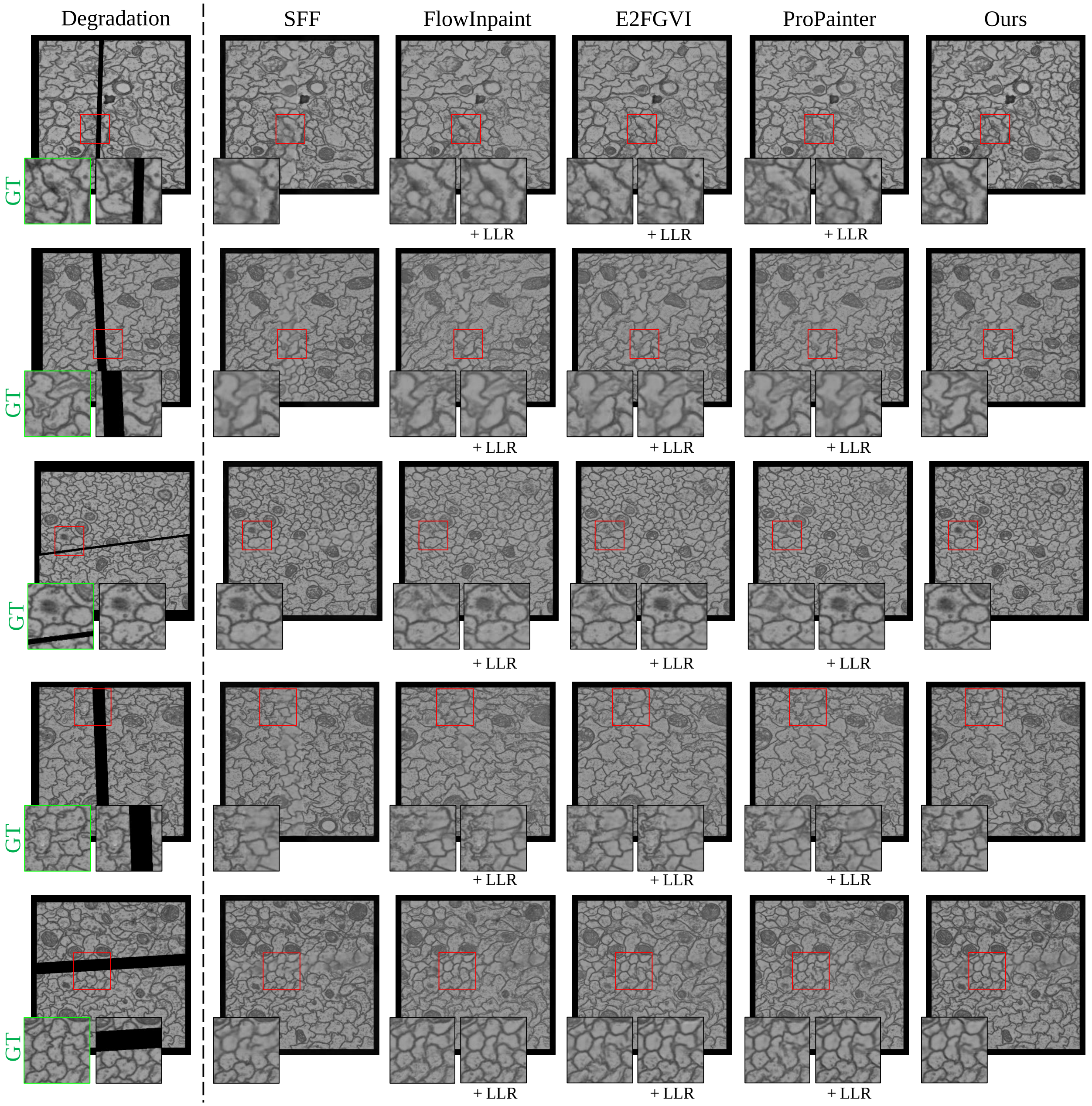

Figure 1. More visual results on the CREMI A dataset.

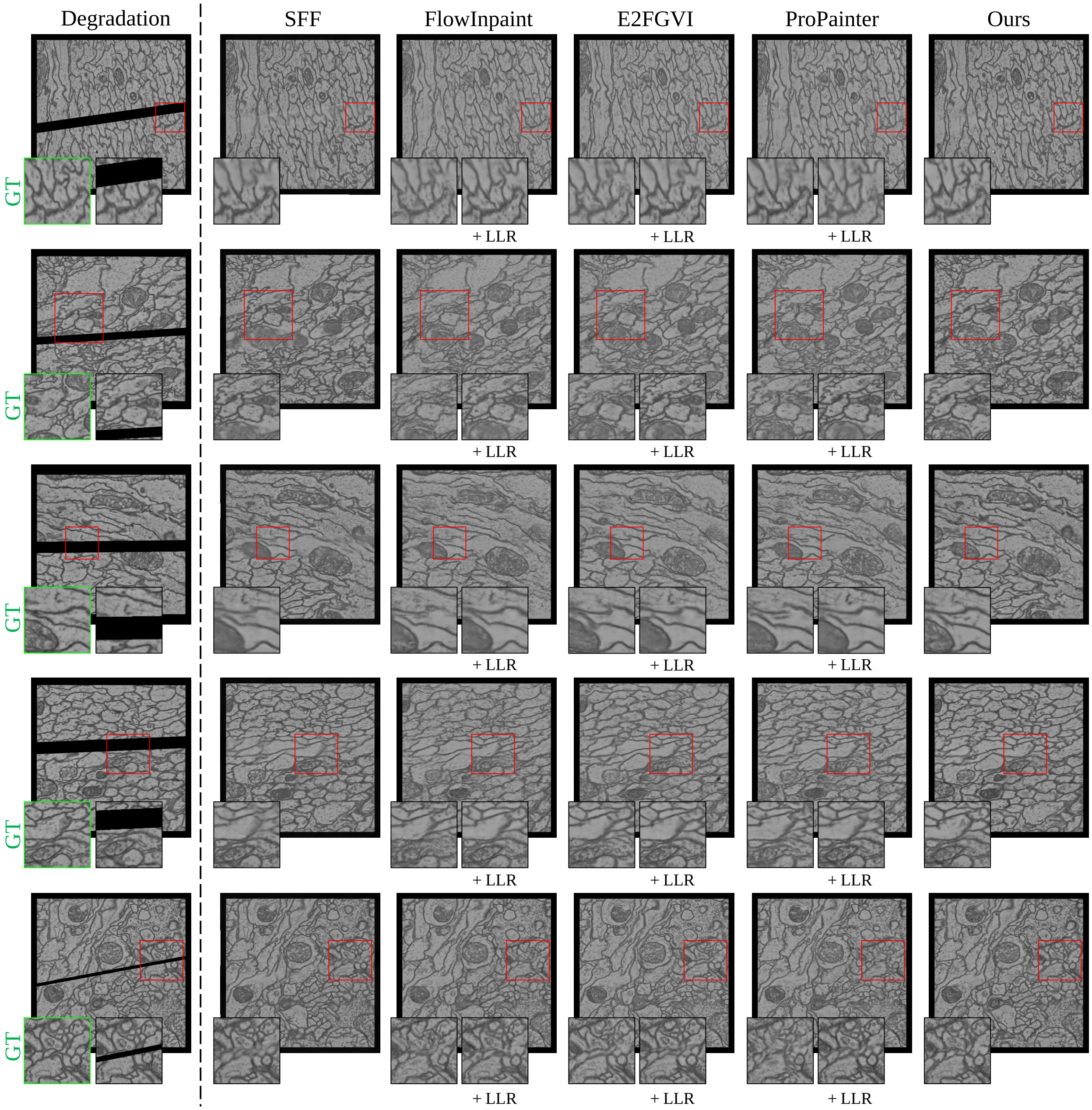

Figure 2. More visual results on the CREMI B dataset.

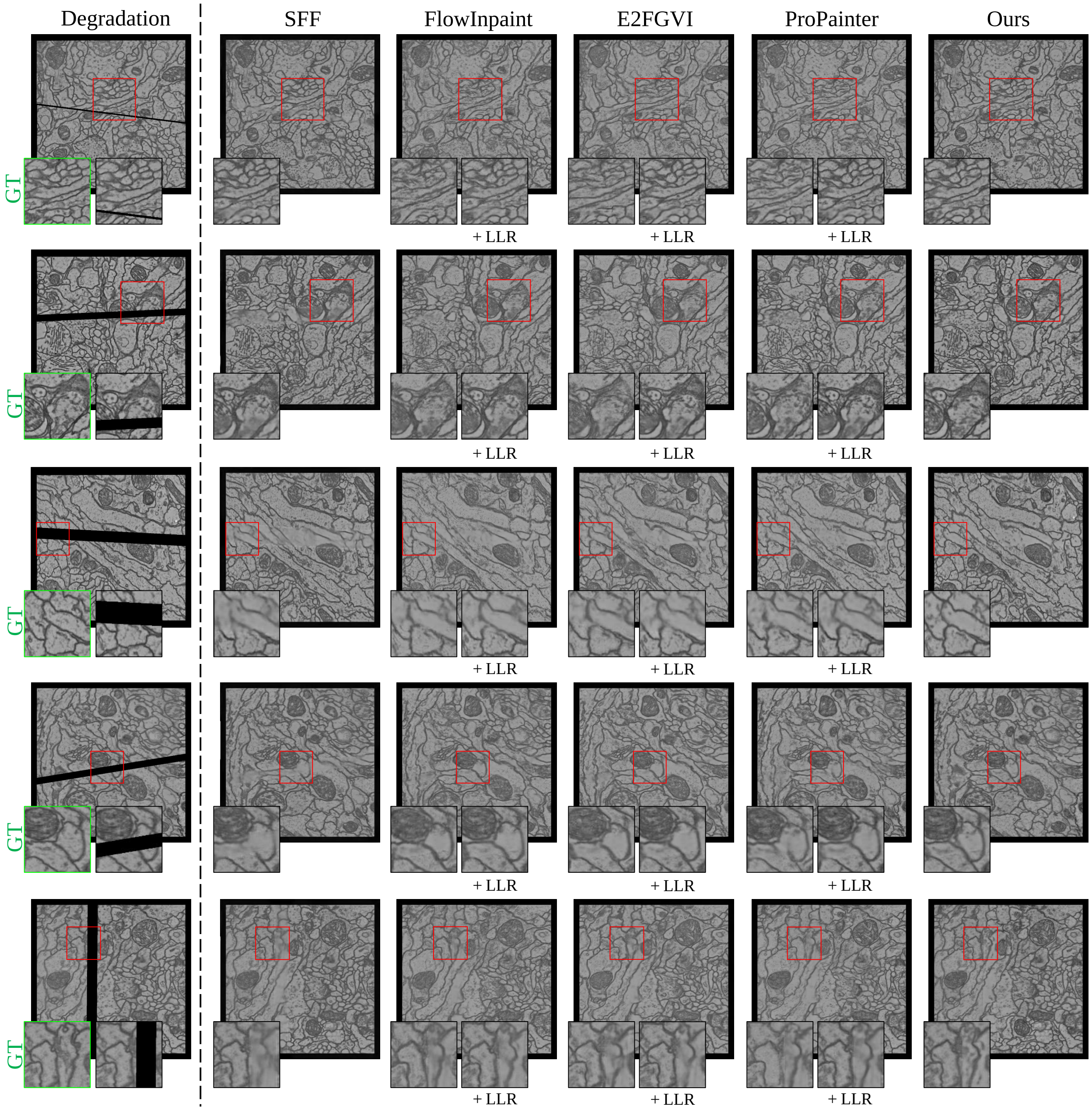

Figure 3. More visual results on the CREMI C dataset.

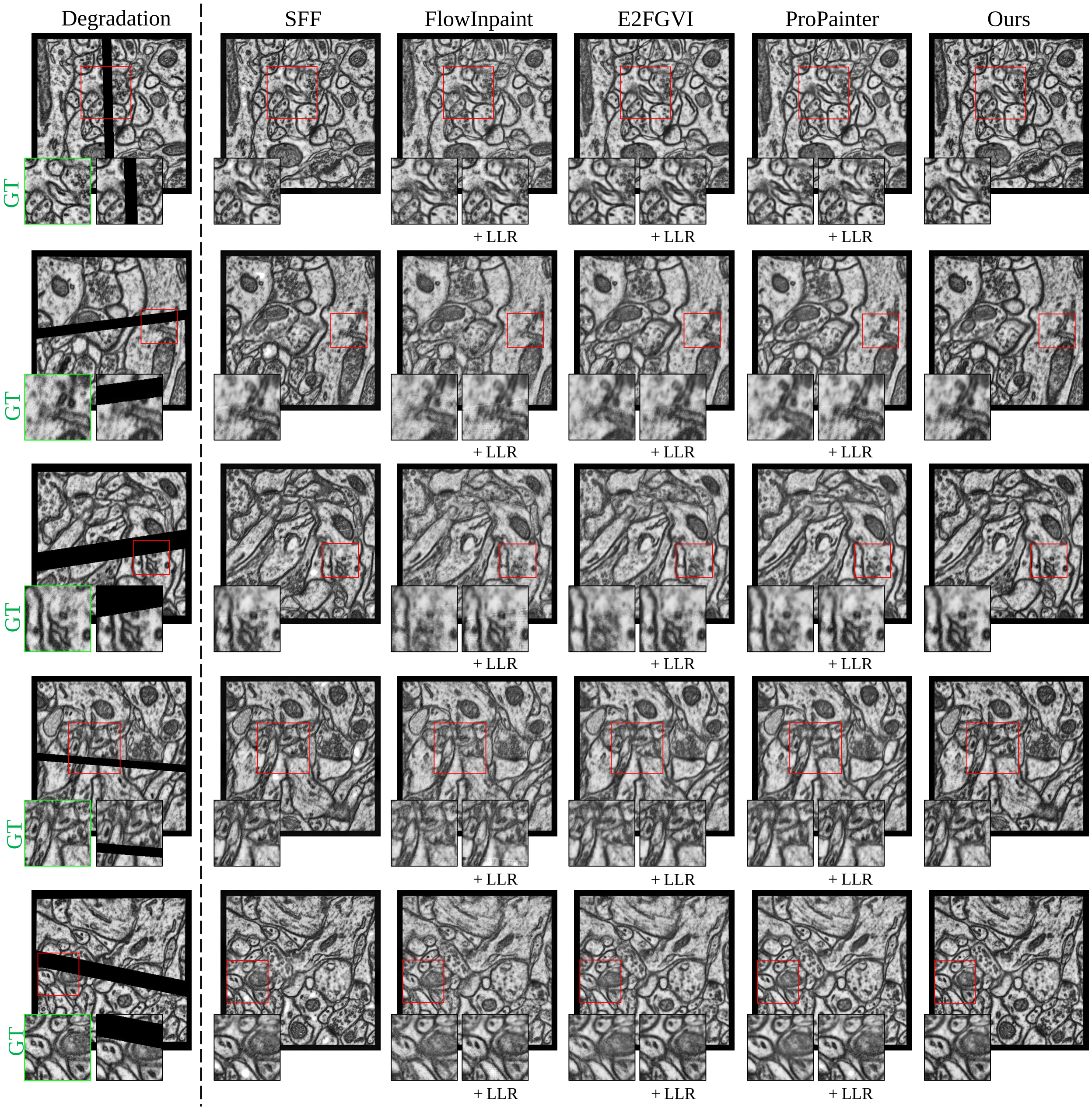

Figure 4. More visual results on the EPFL dataset.

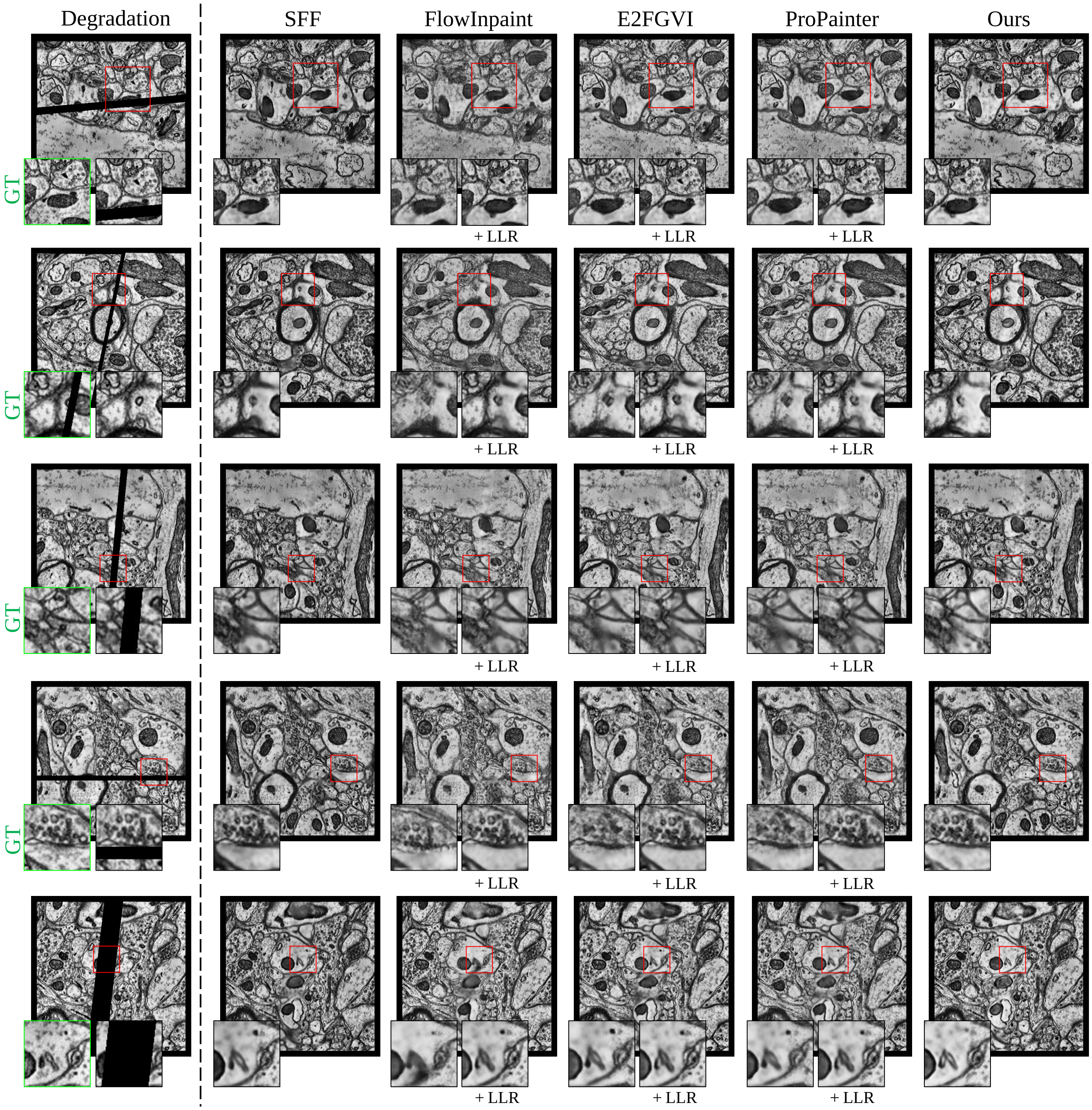

Figure 5. More visual results on the AC3 and AC4 dataset.

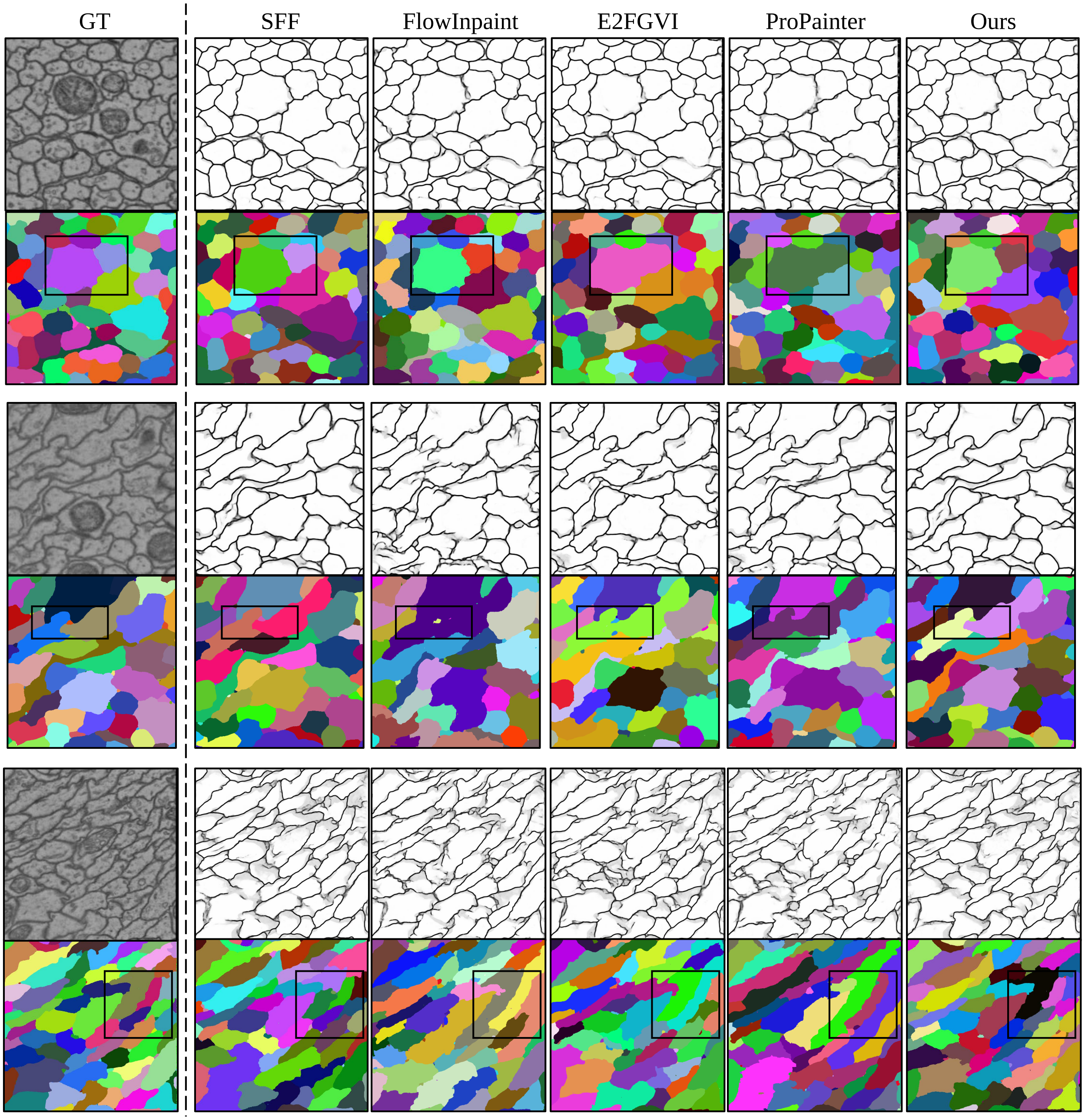

Figure 6. More neuron segmentation results.

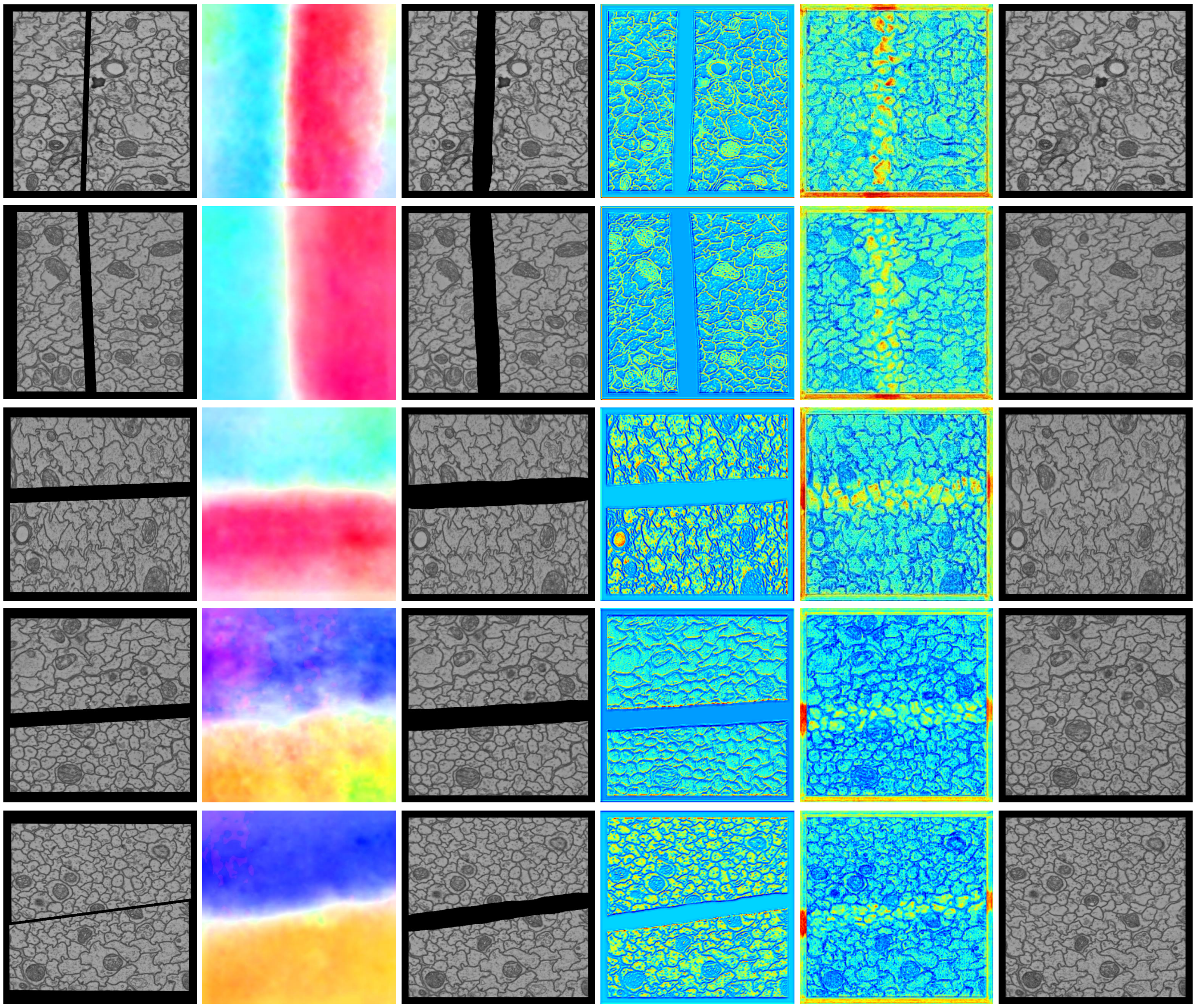

Figure 7. Visualization of deformation fields and intermediate feature maps. (a) SFF degraded image. (b) Displacement field. (c) Unfolded image. (d) Pre-restoration feature map. (e) Post-restoration feature map. (f) Restored image.

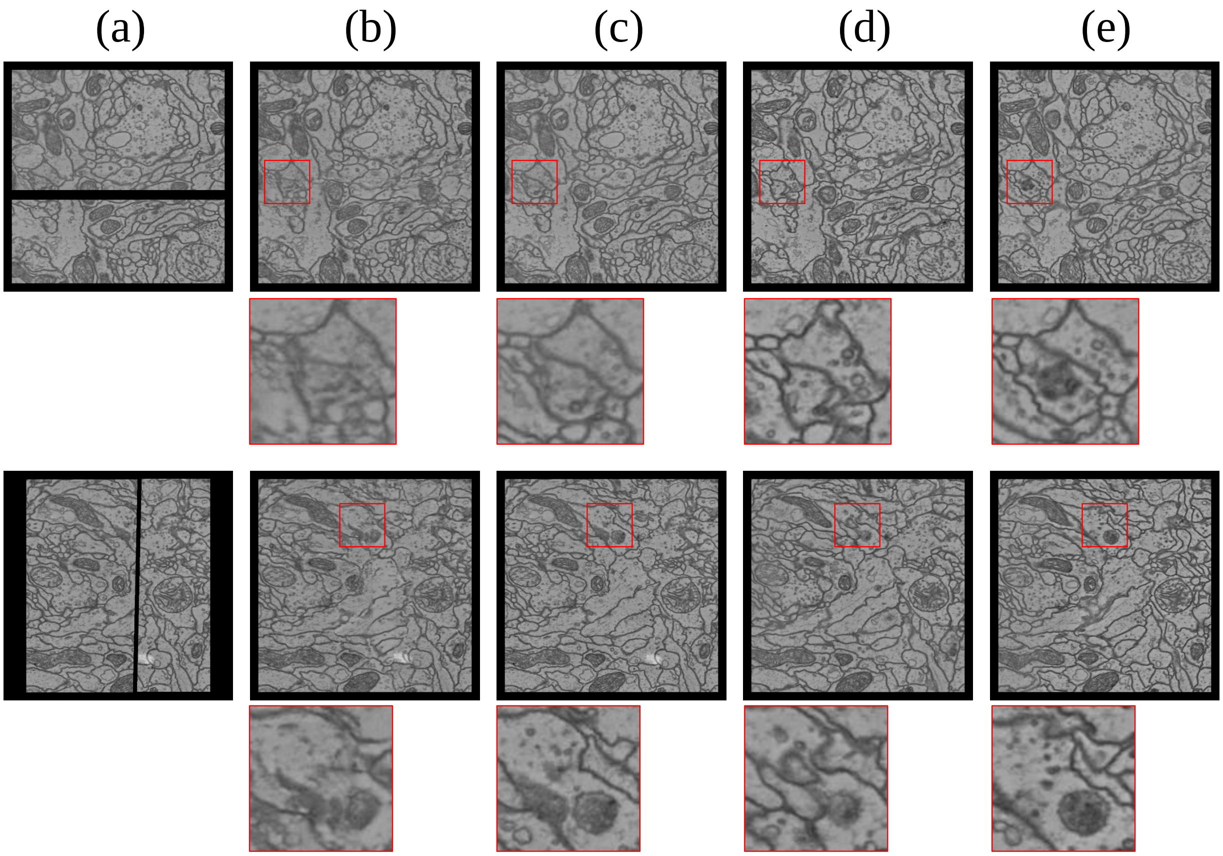

Figure 8. Two failure cases. (a) SFF degraded image. (b) Restored image. (c) Ground truth. (d) Previous slice. (e) Next slice.
